# The COVID-19 Pandemic and Levels of Physical Activity in the Last Trimester, Life Satisfaction and Perceived Stress in Late Pregnancy and in the Early Puerperium

**DOI:** 10.3390/ijerph19053066

**Published:** 2022-03-05

**Authors:** Daria Kołomańska-Bogucka, Agnieszka Micek, Agnieszka I. Mazur-Bialy

**Affiliations:** 1 Department of Biomechanics and Kinesiology, Faculty of Health Science, Jagiellonian University Medical College, Skawińska 8, 31-066 Krakow, Poland; daria.kolomanska@doctoral.uj.edu.pl; 2 Faculty of Health Science, Institute of Nursing and Midwifery, Jagiellonian University Medical College, Kopernika 25, 31-501 Krakow, Poland; agnieszka.micek@uj.edu.pl

**Keywords:** COVID-19, SARS-CoV-2, pregnant women, pregnancy, puerperium, physical activity, life satisfaction, stress, anxiety

## Abstract

Background: The aim of this study was to determine the impact of the COVID-19 pandemic on the levels of physical activity during the third trimester of pregnancy, life satisfaction and stress in women in late pregnancy and early postpartum. Methods: The study was conducted among 740 patients of maternity wards in Cracow hospitals on days 1–8 postpartum. Patients who were surveyed before the pandemic (December 2019–March 2020) were included in the prepandemic group (PPan: *n* = 252). The second group of women (COVID 1 group, Cov1: *n* = 262) was examined in the early stages of the pandemic (May–September 2020). In turn, participants who were surveyed during the population vaccination campaign (June–September 2021) were qualified to the COVID 2 group (Cov2: *n* = 226). The research tools used were the original questionnaire in addition to standardized questionnaires assessing physical activity in the last trimester of pregnancy (the Pregnancy Physical Activity Questionnaire); previous life satisfaction (the Satisfaction with Life Scale); and stress levels during the last month (the Perceived Stress Scale). Results: During the pandemic, women reduced the level of energy spent on total physical activity; nevertheless, statistically significant differences were found only between the PPan and Cov2 groups (*p* = 0.001). At the early stages of the pandemic, patients significantly reduced mobility activities (Cov1 vs. PPan: *p* < 0.001; Cov1 vs. Cov2: *p* = 0.007), while late in the pandemic they spent less energy on household activities (Cov2 vs. PPan: *p* = 0.002, Cov2 vs. Cov1: *p* = 0.002). There were no differences in the levels of stress and life satisfaction. Conclusions: The COVID-19 pandemic impacted the level of physical activity; however, it did not change levels of perceived stress and life satisfaction in women in late-stage pregnancy and in the early puerperium.

## 1. Introduction

Pregnancy is a special period during women’s lives, accompanied by changes, both biological and physiological, as well as psychological and social. These changes may develop from the beginning of pregnancy and even last until the postpartum period [1]. Nausea, vomiting, disruptions with work, frequent medical visits and concerns about the course of pregnancy and childbirth are some of the many causes of anxiety and depression in pregnant women [1,2]. One of the factors that may also affect the mental health of pregnant women is uncertainties related to catastrophic events, which includes the COVID-19 pandemic [3,4]. According to the review by Salari et al. [5], women during the pandemic are at increased risk of developing depression, anxiety and stress. Social isolation makes it difficult to interact with family and friends [4]; hospital visitation restrictions [6] and fear of the possibility of intrauterine transmission of COVID-19 to the fetus [7] pose challenges to the mental health of mothers-to-be [8]. The WHO (World Health Organization) recognizes age, overweight problems and other comorbidities (e.g., hypertension, diabetes) as factors that increase risk of COVID-19 with severe symptomatology for pregnant women [9].

Depressive and anxiety disorders during pregnancy increase the risk of miscarriage [10], preterm labor [11], low birth weight [12] and a low Apgar score (indicates neonatal maladaptation to the intrauterine environment) [13]. On the other hand, high levels of stress in pregnant women may result in the development of emotional, behavioral and cognitive problems in their children [14]. According to a review by Dennis et al. [15], in the first trimester of pregnancy the frequency of anxiety symptoms reported by pregnant women is 18.2%, in the second trimester 19.1% and in the third trimester 24.6%. Furthermore, anxiety can persist after birth of the baby; it is estimated that nearly 15% of postpartum women may have suffered from it. In turn, according to a study conducted during the COVID-19 pandemic for women in perinatal and postpartum periods, anxiety rates ranged from 18 to 56%, while simultaneous anxiety and depressive disorders were experienced by 10 to 33% of women [16]. It has been observed that during the pandemic, there were concerns that COVID-19 could cause fetal structural abnormalities, limit the growth of children or cause preterm births [17]. Similar results were obtained by Ng et al. [18], who also reported higher rates of anxiety in pregnant women resulting from fear of intrauterine transmission of the SARS-CoV-2 virus and its possible consequences.

The negative impact of stress on the quality of life of pregnant women during the COVID-19 pandemic can be minimized by moderate-intensity physical activity [19]. Physical activity before and/or during pregnancy, pregnancy and the postpartum peri-od, and in the postpartum itself reduces the risk of developing and symptoms of perinatal and postpartum depression [20,21]. According to recommendations of the ACOG (The American College of Obstetricians and Gynecologists), a pregnant woman with a normal course should perform moderate-intensity aerobic exercise for at least 150 min per week [22]. Exercise has positive effects on the quality of life, the level of anxiety, stress and fatigue in women in the perinatal and postpartum periods [20,21]. Additionally, many pregnant women try to find positive aspects of the current situation and develop hobbies at home [23].

The aim of this study was to determine the impact of the COVID-19 pandemic on women’s levels of physical activity in the third trimester of pregnancy, their life satisfaction and stress during late pregnancy and early postpartum. In our study, we compared the psychophysical condition of women who gave birth during different periods of the pandemic (December 2019–March 2020, May–September 2020, June–September 2021). To our knowledge, there are currently no publications assessing the psychophysical condition of women who gave birth in Poland during three different periods of the pandemic [24,25,26,27].

## 2. Materials and Methods

### 2.1. Participants

The study was conducted among patients of maternity wards hospitals in Cracow, Poland. The project involved 740 women who were on days 1–8 postpartum (vaginal or cesarean section). Due to the COVID-19 pandemic, women were divided into three groups: prepandemic, COVID 1 and COVID 2. Women in the prepandemic group were studied before the COVID-19 pandemic—from December 2019 to March 2020 (*n* = 252). Seventeen patients refused to participate in the study. Ultimately, 319 questionnaires were distributed. Sixty-five patients returned empty questionnaires and two patients were discharged from the hospital and did not return questionnaires. As a result of restrictions imposed, the project was suspended and resumed in May 2020, in the early stages of the pandemic. Until September 2020, 262 women were examined who were classified as the COVID 1 group. During this period, 379 women were invited to the study, but 22 of them refused to participate due to their lack of time, 93 returned empty questionnaires and 1 patient discharged from the hospital took the questionnaire with her. Additionally, 1 patient was beyond the 8th day of postpartum. The third group of patients was surveyed from June to September 2021 after introduction of population vaccinations against SARS-CoV-2 (COVID 2 group: *n* = 226). In this late stage of the pandemic, 323 patients were enrolled in the study. Fifteen of them immediately refused to participate (the reason—lack of time), while 78 women did not complete any questionnaires at all, and 2 patients discharged from the hospital did not return their questionnaires. Moreover, 2 patients were more than 8 days past the postpartum period.

The inclusion criteria for the study were as follows: age over 18; in the 1st to 8th days of early puerperium; Polish-speaking; and with informed consent given to participate in the study and to data processing. The exclusion criteria were the following: refusal to participate in the study; being under 18 years of age; not Polish-speaking; and being beyond the eighth day of the postpartum period. The study did not establish an upper age limit for the surveyed women. At any time during the survey, patients could resign from further participation without giving any reason.

Participation in the study was voluntary. Informed consent was obtained from all participants. This study was conducted in accordance with the Helsinki Declaration and obtained approval from the Bioethics Committee of Jagiellonian University (Opinion No. 1072.6120.251.2019 of 24 October 2019).

### 2.2. Measures

The research tools used were the original questionnaire (patient’s record) in addition to standardized questionnaires assessing physical activity, life satisfaction and stress levels. The authors’ questionnaire included questions about basic patient information (e.g., age, marital status, education, place of residence), pregnancy and the course of childbirth (e.g., number of deliveries, types of earlier deliveries, complications during childbirth). Physical activity was assessed using the Pregnancy Physical Activity Questionnaire (PPAQ) [28], Polish version [29]. The PPAQ contains 35 questions divided into the following sections: outside work, travel, entertainment and sports and work. On the basis of obtained responses, it is possible to calculate the average weekly energy expenditure for a given activity. Women were asked to specify the amount of time spent on an activity (depending on the question: 0–6 h or more per day, 0–3 h or more per week) [28,29,30]. The level of satisfaction with life was determined on the basis of the Satisfaction with Life Scale (SWLS), using the Polish version adapted by Juczyński Z. [31]. The SWLS scale consists of 5 statements, which are rated on a scale from 1 (‘completely disagree’, low life satisfaction) to 7 (‘completely agree’, high life satisfaction). The sum of points from all statements determines the so-called general index of satisfaction with life. The score range is between 5 and 35 points. The higher the total score obtained in the SWLS, the higher the satisfaction with life. Cronbach’s alpha reliability index for the SWLS is 0.81 (Polish version) [31,32]. The SWLS scale is a frequently used scale to assess life satisfaction in perinatal women [33,34,35,36,37]. Stress was assessed on the basis of the Perceived Stress Scale (PSS-10), using the Polish version adapted by Juczyński and Ogińska-Bulik [38]. The PSS-10 contains 10 questions on subjective feelings about problems, personal events and behavior in stressful situations during the past month. Each question is rated on a scale from 0 (‘never’) to 4 (‘very often’), and the final score is between 0 and 40 points. The higher the total score obtained by the patient, the higher the level of perceived stress. Cronbach’s alpha reliability index for the PSS-10 is 0.86 (Polish version) [38,39]. The PSS-10 scale can be used to assess the perceived stress in women during the perinatal period [8,24,25].

### 2.3. Statistical Analysis

A priori, the required sample size was calculated. We assumed a Type I error of 0.05 and conservatively set the power at a very high level of 95%. Additionally, we allowed the possibility of using non-parametric tests by incorporating the Pitman ARE (asymptotic relative efficiency) correction. Under such circumstances, we estimated that 723 persons were sufficient to detect differences of a small effect size (0.15) in the distribution of total physical activity among three groups of roughly equal sizes.

The PS Imago Pro 7 program and R software version 4.0.2 (Development Core Team, Vienna, Austria) were used for statistical analyses. Normality of the obtained data was assessed using the Shapiro–Wilk test. The Kruskal–Wallis test followed by the Wilcoxon rank-sum test with Bonferroni correction for pairwise comparisons were applied to check differences among three independent groups and to identify which pairs differed. A chi-square test of independence was performed to evaluate the relationship between two qualitative variables. Correlations between total physical activity and its components were estimated with Spearman rank correlation coefficients. A multivariable linear regression analysis was used to find factors which influenced energy expenditure on total physical activity and its domains. Possible confounders were identified based on the literature. For each subscale and for physical activity in total, the separate models were fitted with different sets of covariates included to check robustness of the findings. Unstandardized β-coefficients were presented as size effects reflecting the associations. A value of *p* < 0.05 was considered significant.

## 3. Results

Detailed characteristics of the study group are presented in Table 1. In all three groups the median age of participants was 31 years. In all groups, the majority of women were married, lived in large cities and had higher education. There were also higher percentages of vaginal deliveries than caesarean sections, but the differences were not statistically significant. It was observed that in the prepandemic and COVID 2 groups more women were primiparous, while in the COVID 1 group more women were multiparous. Regardless of the examination period, most women planned to become pregnant in their current pregnancies and their deliveries were on time.

### 3.1. Physical Activity

During hospital stays patients characterized physical activities undertaken in their third trimesters of pregnancy (PPAQ questionnaire). The study showed that the energy spent by women in the third trimester of pregnancy on total physical activity was significantly lower in postpartum women from the COVID 2 group compared to those in the prepandemic group (Me: 146.5, Q1–Q3: 95.1–202.6 MET vs. Me: 170.7, Q1–Q3: 114.1–234.6 MET). Similar differences were not found among participants giving birth in the early part of the pandemic and participants who delivered before or in the late stage of the pandemic. In contrast, women who gave birth between May and September 2020 spent less energy on movement-related activities compared to women from the prepandemic (Me: 8.8, Q1–Q3: 3.4–18.8 MET vs. Me: 14.0, Q1–Q3: 6.7–26.1 MET) and COVID 2 groups (Me: 8.8, Q1–Q3: 3.4–18.8 MET vs. Me: 12.6, Q1–Q3: 5.3–22.6 MET). However, as the pandemic continued, women stopped limiting their activities related to movement. Both prepandemic and late-stage participants spent similar amounts of energy on this type of activity (Me: 14.0, Q1–Q3: 6.7–26.1 MET for prepandemic group vs. Me: 12.6, Q1–Q3: 5.3–22.6 MET for the COVID 2 group). Nevertheless, in the late stage of the pandemic, women spent less energy on domestic activities (Me: 71.1, Q1–Q3: 34.7–132.3 MET) than women giving birth before (Me: 93.5, Q1–Q3: 57.8–141.5 MET) or in the early time of the pandemic (Me: 95.9, Q1–Q3: 46.8–154.0 MET). The detailed characteristics of the obtained results are presented in Table 2.

Most components of physical activity significantly correlated with total physical activity, with the strongest effect observed for household activities (r = 0.786, *p* < 0.001), occupational activities (r = 0.584, *p* < 0.001) and movement (r = 0.497, *p* < 0.001). However, the correlations between particular domains were rather weak and estimated at a level below 0.278 on absolute value. Most of the pairwise comparisons showed positive associations except household activities, which correlated negatively with passive rest (r = −0.261, *p* < 0.001). The correlation results are presented in Table 3.

### 3.2. Levels of Life Satisfaction and Stress in Women

Life satisfaction was assessed using the SWLS scale categorized into low (values 1–4 sten), average (5–6 sten scores) and high (7–10 sten) [31]. Regardless of the period of the study, most of the patients felt high satisfaction with their lives (prepandemic: 66.3%, COVID 1: 70.0% and COVID 2: 67.4% of women in each group). Nevertheless, no statistically significant differences in the degree of life satisfaction were observed among the women studied. The level of perceived stress was also characterized in the patients. As a result of the fact that the PSS-10 stress scale is a sten scale, the levels of stress were also divided into low (1–4 sten scores), moderate (5–6 sten values) and high (7–10 sten scores) [38]. The distributions of stress levels in all studied groups were similar. The obtained results of descriptive and test statistics are presented in Table 4.

However, both in participants giving birth before, as well as in the early and late periods of the COVID-19 pandemic, it was observed that with increasing life satisfaction, the level of perceived stress decreased. Nevertheless, in the groups studied during the pandemic the correlation coefficients were weak (COVID 1 group: r = −0.385, *p* < 0.001; COVID 2 group: r = −0.331, *p* < 0.001). A stronger correlation between life satisfaction and perceived stress was demonstrated among women examined before the pandemic (r = −0.420, *p* < 0.001). Results of the conducted correlations are presented in Table 5.

### 3.3. Levels of Physical Activity and Life Satisfaction

Correlations were made between the parameters of physical activity and life satisfaction. A negative correlation between the degree of life satisfaction and the amount of energy devoted to passive rest was observed among patients from the prepademic group (r = −0.153, *p* = 0.024). No similar correlations were found between the other variables among all groups (Table 6).

### 3.4. Physical Activity and the Level of Perceived Stress

The analysis of individual components of physical activity in relation to the level of perceived stress by women from all groups was also performed. It should be noted that in women giving birth before the pandemic, higher levels of stress correlated with lower energy expenditure on sports activities (r = −0.134, *p* = 0.048). On the other hand, among participants giving birth at the initial stage of the pandemic, a positive correlation was observed between the level of stress and the amount of energy spent on domestic activities (r = 0.149, *p* = 0.026). No similar relationships were found between other types of physical activity and the level of stress—Table 7.

### 3.5. Level of Physical Activity—Multivariable Linear Regression Analysis

Multivariable linear regression analysis was performed to check associations between total physical activity and its subdomains during the period of pandemic, SWLS, stress and other factors after controlling for other confounders. The analysis revealed that independently of the remaining covariates, women from the COVID 1 and COVID 2 groups had significantly lower levels of physical activity in total, as well as in household activities, compared with the prepandemic group (β = −17.7, 95% CI: −35.2 to −0.3 and β = −14.4, 95% CI: −27.1 to −1.8 for COVID 1 compared with the prepandemic group; β = −26.1, 95% CI: −43.7 to −8.5 and β = −24.1, 95% CI: −36.8 to −11.4 for COVID 2 compared with the prepandemic group, respectively, in fully adjusted models). Moreover, a decreasing trend across considered groups was observed with the strongest decline for women who gave birth during the late stage of the pandemic (COVID 2 group). Additionally, a decrease in movement for women during the first period of the pandemic (COVID 1 group) was detected; however, later in the pandemic movement assessment results stabilized with no differences between the COVID 2 and prepandemic groups found. One unit increase in score of overall satisfaction from life was associated with lower energy expenditure on passive rest (β = −0.6, 95% CI: −0.9 to −0.2 in Model 3). The results for stress did not show any stable and robust relationship with any domain of physical activity. However, in model 1 it was noted that as perceived stress increased by one unit, participants increased energy expenditure on total physical activity (β = 1.3, 95% CI: 0.1 to 2.5) and household activities (β = 1.1, 95% CI: 0.2 to 2.1). However, unmarried women devoted much more energy to total activity, as well as to passive rest, movement, household activities and activities related to work (β = 42.4, 95% CI: 20.3 to 64.6; β = 4.6, 95% CI: 0.0 to 9.2; β = 7.7, 95% CI: 2.9 to 12.6; β = 14.9, 95% CI: 0.3 to 29.6; and β =23.4, 95% CI: 5.9 to 40.9, respectively). On the other hand, women who lived in villages and in cities (up to 100,000 inhabitants) spent significantly less energy on passive rest (β = −5.8, 95% CI: −9.4 to −2.3 and β = −4.9, 95% CI: −9.8 to −0.1, respectively) and more on home activities (β = 14.3, 95% CI: 2.0 to 26.6 and β = 17.8, 95% CI: 0.9 to 34.7). In addition, participants living in villages had lower energy expenditures on sports activities compared to women living in large cities (β = −2.9, 95% CI: −4.8; −0.9). It was also observed that regardless of other covariates, multiparous women had significantly higher levels of total physical activity (β = 53.7, 95% CI: 37.8 to 69.6) and household activities (β = 74.7, 95% CI: 63.2 to 86.2) compared to primiparaous women. However, multiparous women spent less energy on sports activities (−2.2, 95% CI: −4.0 to −0.4) and passive rest (−14.7, 95% CI: −18.0 to −11.3). The exact results of the regression analysis are shown in Table 8.

## 4. Discussion

The aim of this study was to determine the impact of the COVID-19 pandemic on the levels of physical activity during the third trimester of pregnancy, life satisfaction and perceived stress in women in late pregnancy and in early postpartum during the period from December 2019 to September 2021. In our study, we observed that both women who gave birth in the initial and late stages of the pandemic decreased their level of energy spent on total physical activity compared to women giving birth before the pandemic. We did not notice similar differences in levels of perceived stress and life satisfaction.

In recent years, physical activity during pregnancy has been recommended as a factor supporting the physiological course of pregnancy and childbirth [40]. Due to the many benefits of regular activity during the perinatal period, pregnant women often modify their previous behavior and adapt to principles of a healthy lifestyle [41]. Nevertheless, pain, fear of harming the baby and of miscarriage or premature birth, in addition to fear of suffering an injury and lacking sufficient knowledge, are some of the most commonly reported reasons for not being active during pregnancy [42,43]. Changes in the level of physical activity may also be caused by the development of anxiety and depressive disorders [44], which may worsen as a result of the current pandemic state [45]. After the announcement of the pandemic and introduction of restrictions, many pregnant or postpartum women reduced their previous physical activity [46].

In our study, we showed that women who gave birth in any period of the pandemic spent less energy on total physical activity in the third trimester of pregnancy (prepandemic Me: 170.7, Q1–Q3: 114.1–234.6 MET; COVID 1 group Me: 150.8, Q1–Q3: 103.0–229.5 MET; and COVID 2 group Me: 146.5, Q1–Q3: 95.1–202.6 MET). For comparison, in a study conducted in 2017 which also considered Polish women in the third trimester of pregnancy, the median energy expenditure for total physical activity was 196.9 MET (Q1–Q3: 137.1–268.9 MET) [47]. In turn, Krzepota el at. [30] observed that when examining the same pregnant women in a one-week time interval, the energy expenditure for total activity could decrease from 214.64 MET to 192.35 MET (Q1–Q3: 172.08–274.38 and 149.24–248.93 MET, respectively). Other studies conducted among women in their second and third trimesters showed that levels of total physical activity regardless of the period of pregnancy were similar and amounted to 166.8 MET (Q1–Q3: 129.1–220.7 MET) and 168.8 MET (Q1–Q3: 130.4–227.2 MET), respectively [48]. Comparing our results with the results obtained by other researchers, it can be observed that during the pandemic, women had lower overall physical activity. Based on linear regression analysis of many variables, we showed that regardless of the remaining covariates, women giving birth during the early and late stages of the pandemic had significantly lower levels of overall physical activity compared to women giving birth before the pandemic. Moreover, this observed decrease was stronger for women in the COVID 2 group (late stage of pandemic).

We furthermore noted that in each study group, out of all types of activity, participants spent the most energy on household activities. Similar results were also obtained in other studies [30,47,48,49,50]. Based on our results of the linear regression analysis, it can be seen that regardless of coexisting factors, pregnant women significantly reduced activities related to movement during the early stage of the pandemic. Similar relationships were not shown in the late period of the pandemic. This difference could be a result of current restrictions, the state of knowledge about virus transmission and the course of infection in pregnant women. On the other hand, there were no significant changes in the levels of energy devoted to passive rest among the surveyed women. Only women from the COVID 2 group, irrespective of life satisfaction and stress, decreased their expenditure on passive activity compared to the prepandemic group. However, no similar relationships were found in the remaining regression models (2 and 3) as well as in the group of women who gave birth in the early stage of the pandemic. Research by Meaney et al. [51] indicated that women most often try to reduce stress through contact with other people (e.g., talking to their husband/partner, family, friends) and through physical exercise (e.g., walking, yoga, unspecified exercises). Biviá-Roig et al. [52] observed a decrease in overall physical activity in pregnant women, which was associated with an increase in time spent in sitting positions (it doubled during the pandemic). In turn, Lebel et al. [53] noted that pregnant women in the early stage of the pandemic were characterized by good physical activity levels. In addition, physical activity reduced psychological symptoms.

A pandemic can have both positive and negative effects on lifestyle. In the study by Hori et al. [54] there were no significant changes in physical activity levels resulting from lifestyle changes during the COVID-19 pandemic for both primaparous and multiparous women. There were moreover no differences in physical activity levels between women who said the pandemic had affected their current activity and those who reported that the pandemic had not affected them. In contrast, our study showed that multiparous women spent more energy on total physical activity and household activities, and significantly less on sports and passive rest compared to primiparous women. Kumari et al. [55] showed that the pandemic changed the daily routines of women during perinatal and postnatal periods, e.g., women reported that they were unable to maintain regular physical activity and a proper diet. Other studies indicate that the percentage of pregnant women leading a mainly passive lifestyle during the pandemic was 79%, with only 23% of those who were active following guidelines for physical activity during pregnancy (before the pandemic, this percentage was 47%). Fear of leaving home due to the prevalence of COVID-19 was the most commonly reported reason for a decline in activity [56]. Muhaidat et al. [57] noted that during pregnancy as much as 35.91% of women decreased their physical activity compared to the period before the lockdown. There were 6.04% of women who maintained their current level of activity, while 2.01% increased it. A reduction of physical activity during the pandemic in pregnant women (regardless of the trimester) was also noted in studies by Hashim et al. [58]—of all surveyed women, 53.6% reduced time spent on physical activity and only 13.8% increased it. In turn, Hu et al. [59] showed that during the early stage of the pandemic, more nonpregnant women reduced their physical activity compared to pregnant women (50.0% vs 40.4%). Nascimento el al. [50] indicated that higher education, primiparity, work, medical private care, pregnancy planning, pre-pregnancy activity and exercise information/tips had a significant impact on the level of physical activity in pregnancy.

Pregnancy should be a time of emotional well-being in a woman’s life; unfortunately, for many women it is a time full of fear, anxiety, increased stress and even the development of depressive disorders [60]. Among the participants in our study, we observed a negative correlation between perceived stress levels and life satisfaction. In addition, most women in each group (over 60%) reported high satisfaction with their lives. However, our study showed that regardless of the period of delivery, the level of overall stress experienced by women remained unchanged (prepandemic group Me: 18.0, Q1–Q3: 13.0–22.0 points; COVID 1 group Me: 17.0, Q1–Q3: 13.0–22.0 points; and COVID 2 group Me: 17.0, Q1–Q3: 13.0–22.0 points). In addition, the percentages of women with low, medium and high stress were similar when comparing them within and between groups. In contrast, Puertas-Gonzalez et al. observed that women who were pregnant during the pandemic had a higher level of perceived stress than women who were pregnant before the pandemic [61]. Available studies indicate that women who consider COVID-19 as a stress factor experience higher levels of stress than pregnant women who do not link it to their current situation [8]. Stepowicz et al. [25] showed that levels of stress and anxiety in pregnant or postpartum women during the pandemic were moderate to high. In other studies conducted in Poland during the pandemic, a moderate and positive relationship among women was observed between perceived stress, fear of COVID-19 and fear of childbirth [24]. Ilska et al. [26] noted that during the second wave of the pandemic, almost 38.5% of pregnant women reported high preparedness stress and 26% reported high perinatal infection stress. In turn, Jiang et al. [62] observed that in China in February 2020 the prevalence of perceived stress in pregnancy was 89.1%. Other studies also show that during the pandemic, women report high levels of stress associated with both pregnancy and the COVID-19 pandemic [63]. According to a review by Campos-Garzón et al. [64] the level of stress experienced by pregnant women is influenced by feelings of loneliness and fear of infection. Moreover, stress has been recognized as a predictor of anxiety and depression symptoms related to COVID-19. Meta-analysis performed by Demissie et al. [65] estimates that as much as 56% of pregnant and breastfeeding women experienced stress during the pandemic. The surveyed women also had depressive disorders (27%), anxiety (33%), sleep problems (34%) and social dysfunction (24%). Other studies have also shown that at the beginning of the pandemic, women’s anxiety about their own, their child’s and their partner’s health increased drastically. Similar changes were also noted in the partners of pregnant women [66]. In turn, Çiler Eren et al. [67] observed that a diagnosis of COVID-19 in a pregnant family resulted in high scores in all cases, yet had no effect on the overall scores for depression, stress and anxiety. Nevertheless, there are many studies which show that pregnant women were exposed to stressful situations before the pandemic was announced [68,69,70,71,72]. Low socioeconomic status, lack of support from family, violence from partners and obstetric factors including unplanned pregnancy have been identified as socio-demographic factors having significant impacts on negative mental health changes in pregnant women [73].

### Strengths and Limitations

There are several strengths and limitations of this study. The strength of this study comes from surveying women during different stages of the pandemic; this provides us a look at whether or not the period of the pandemic (which differed in knowledge about the virus, restrictions and vaccination possibilities) had an impact on particular aspects of women’s behavior. One limitation of this study is that women in the prepandemic group were tested from December 2019 to March 2020 when no pandemic state was yet declared, even as the virus was already present in China and in other countries. Moreover, we only examined women who were patients in Cracow hospitals. The introduced restrictions, numbers of cases and vaccinations differed between particular areas in Poland, therefore we cannot compare our results to the general population of women in the perinatal period. Moreover, we assessed levels of activity, perceived stress and satisfaction with life using self-report questionnaires which may bias the obtained data. Therefore, longitudinal studies with multiple points of data collection are needed to better understand patterns of change in the psychological states of women. In addition, testing should be extended throughout pregnancy and the postpartum period.

## 5. Conclusions

It was observed that women in the third trimester of pregnancy during the pandemic reduced their levels of energy spent on total physical activity compared to pregnant participants before the pandemic. The scope and types of activities may have been influenced by the introduced restrictions that limited, for example, access to parks, forests and fitness clubs. The introduction of restrictions related to spending free time outside the home, learning and remote work could cause changes in the performance of household duties by individual household members. In the research it was noted that women who were pregnant in the later stage of the pandemic spent significantly less energy on home activities. Women who gave birth in 2020 (COVID 1 group) also reported significantly reduced levels of activity related to movement. There were no significant changes in the levels of life satisfaction and perceived stress in late pregnancy and early postpartum among the examined patient groups. Most women (regardless of the period of the study) had a high level of life satisfaction. Both before and during the pandemic (early and late period), the distribution of the level of perceived stress was similar. It was also noted in all groups that as life satisfaction increased, stress decreased. Nevertheless, the strongest correlation between stress and life satisfaction was found among women giving birth before the pandemic. The individual parameters of stress and life satisfaction affected the levels of certain physical activity components in the surveyed women.

## Figures and Tables

**Table 1:** Characteristics of the studied groups.

Parameters	Prepandemic Group (*N* = 252)	COVID 1 Group (*N* = 262)	COVID 2 Group (*N* = 226)	*p* *
Age [Me (Q1; Q3)]	31.0 (27.8; 33.3)	31.0 (28.3; 34.0)	31.0 (28.0; 33.8)	0.110
Marital status [*n*/%]				0.079
Single	9/3.6	9/3.4	5/2.2	
A partnership	41/16.3	27/10.3	20/8.8	
Married	202/80.1	226/86.3	201/88.9	
Place of residence [*n*/%]				0.330
Village	59/23.4	72/27.5	54/23.9	
City < 100,000	25/9.9	26/9.9	33/14.6	
City > 100,000	168/66.7	164/62.6	139/61.5	
Education [*n*/%]				0.359
Primary/vocational/secondary	48/19.1	61/23.3	42/18.6	
University	203/80.9	201/76.7	184/81.4	
Type of delivery [*n*/%]				0.105
Vaginal delivery	149/63.7	152/58	122/54.0	
Cesarean section	85/36.3	110/42	104/46.0	
Number of deliveries [*n*/%]				0.020
Primiparous	137/54.4	111/42.4	115/50.9	
Multiparous	115/45.6	151/57.6	111/49.1	
Number of pregnancies [Me (Q1; Q3)]	1 (1; 2) ^a,#^	2 (1; 3) ^a,#^	2 (1; 2)	0.002
Planning the current pregnancy [*n*/%] Yes	192/76.2	218/83.2	185/81.9	0.108
Complications in pregnancy [*n*/%] Yes	44/19	35/13.4	43/19	0.152
Delivery at term [*n*/%] Yes	140/59.8	162/61.8	126/55.8	0.386

Me—median; Q1—lower quartile; Q3—higher quartile; prepandemic group—participants tested before the pandemic (December 2019 to March 2020); COVID 1 group—participants tested in the early stages of the pandemic (May to September 2020); COVID 2 group—participants tested in the late stages of the pandemic (June to September 2021); ^#^ the same letter denotes significant difference between designated groups: ^a^—prepandemic vs. COVID 1. * based on Kruskal–Wallis test followed by pairwise comparisons using Wilcoxon rank-sum test with Bonferroni correction or chi-square test of independence.

**Table 2:** The Level of Physical Activity in the Studied Women.

	Prepandemic Group	COVID 1 Group	COVID 2 Group	
Physical Activity [MET-h/Week]	(*N* = 220 ^&^) Me (Q1; Q3)	(*N* = 244 ^&^) Me (Q1; Q3)	(*N* = 223 ^&^) Me (Q1; Q3)	*p* *
Total physical activity	170.7 (114.1; 234.6) ^b,#^	150.8 (103.0; 229.5)	146.5 (95.1; 202.6) ^b,#^	0.007
Sports activity and physical exercise	3.2 (0.9; 11.0)	4.5 (1.6; 12.1)	3.9 (1.6; 10.8)	0.206
Passive rest	20.4 (12.8; 41.0)	17.8 (8.9; 35.7)	20.0 (8.9; 34.5)	0.106
Movement	14.0 (6.7; 26.1) ^a,#^	8.8 (3.4; 18.8) ^a,c,#^	12.6 (5.3; 22.6) ^c,#^	<0.001
Household activities	93.5 (57.8; 141.5) ^b,#^	95.9 (46.8; 154.0) ^c,#^	71.1 (34.7; 132.3) ^b,c,#^	0.001
Occupational activity	57.8 (23.2; 76.9)	52.9 (22.4; 78.2)	67.1 (32.5; 74.3)	0.979

Me—median; Q1—lower quartile; Q3—higher quartile; prepandemic group—participants tested before the pandemic (December 2019 to March 2020); COVID 1 group—participants tested in the early stages of the pandemic (May to September 2020); COVID 2 group—participants tested in the late stages of the pandemic (June to September 2021); ^#^ the same letter denotes significant difference between designated groups: ^a^—prepandemic vs. COVID 1, ^b^—prepandemic vs. COVID 2, ^c^—COVID 1 vs. COVID 2; MET-h/week—metabolic equivalent of task (average weekly energy expenditure: MET-hour/week); * based on Kruskal–Wallis test followed by pairwise comparisons using Wilcoxon rank-sum test with Bonferroni correction; ^&^ for work-related activities: prepandemic group *N* = 67, COVID 1 group *N* = 58, COVID 2 group *N* = 63.

**Table 3:** Spearman’s Rank Correlations between Total Physical Activity [MET-h/Weeks] and its Components.

Lp	Physical Activity	1	2	3	4	5	6
1	Total physical activity	1.000					
2	Sports activity and physical exercise	0.244 ***	1.000				
3	Passive rest	0.118 **	0.081 *	1.000			
4	Movement	0.497 ***	0.210 ***	0.096 *	1.000		
5	Household activities	0.786 ***	0.107 **	−0.261 ***	0.278 ***	1.000	
6	Occupational activity	0.584 ***	0.040	0.230 **	0.207 **	−0.005	1.000

*** *p* < 0.001, ** *p* < 0.01, * *p* < 0.05

**Table 4:** Levels of Life Satisfaction and Perceived Stress of Women.

Parameters	Prepandemic Group	COVID 1 Group	COVID 2 Group	*p* *
	(*N* = 252)	(*N* = 262)	(*N* = 226)	
SWLS [Me (Q1; Q3)]	26.0 (23.0; 29.0)	26.0 (23.0; 29.0)	25.0 (22.0; 28.0)	0.625
SWLS [*n*/%]				0.571
Low	16/6.6	8/3.3	12/5.4	
Moderate	66/27.2	65/26.7	60/27.1	
High	161/66.3	170/70.0	149/67.4	
Stress [Me (Q1; Q3)]	18.0 (13.0; 22.0)	17.0 (13.0; 22.0)	17.0 (13.0; 22.0)	0.974
Stress [*n*/%]				0.902
Low	68/27.9	70/29.3	58/26.6	
Moderate	81/33.2	85/35.6	78/35.8	
High	95/38.9	84/35.1	82/37.6	

Me—median; Q1—lower quartile; Q3—higher quartile; prepandemic group—participants tested before the pandemic (December 2019 to March 2020); COVID 1 group—participants tested in the early stages of the pandemic (May to September 2020); COVID 2 group—participants tested in the late stages of the pandemic (June to September 2021); * based on Kruskal–Wallis test or chi-square test of independence.

**Table 5:** Spearman’s Rank Correlations between the Levels of Life Satisfaction and Perceived Stress.

Group	Prepandemic Group		COVID 1 Group		COVID 2 Group	
Parameters	*p*	Spearman’s Rank	*p*	Spearman’s Rank	*p*	Spearman’s Rank
SWLS vs. PSS-10	<0.001	−0.420	<0.001	−0.385	<0.001	−0.331

Statistical significance for *p* < 0.05; Spearman’s rank—Spearman’s rank correlation coefficient; prepandemic group—participants tested before the pandemic (December 2019 to March 2020); COVID 1 group—participants tested in the early stages of the pandemic (May to September 2020); COVID 2 group—participants tested in the late stages of the pandemic (June to September 2021).

**Table 6:** Spearman’s Rank Correlations—Physical Activity and Life Satisfaction.

Satisfaction of Life						
Group	Prepandemic Group		COVID 1 Group		COVID 2 Group	
Parameters	*p*	Spearman’s Rank	*p*	Spearman’s Rank	*p*	Spearman’s Rank
Total physical activity	0.480	0.048	0.601	−0.035	0.291	−0.072
Sports activity and physical exercise	0.177	0.092	0.174	0.091	0.333	0.066
Passive rest	0.024	−0.153	0.114	−0.105	0.400	−0.057
Movement	0.747	−0.022	0.663	−0.029	0.340	−0.065
Household activities	0.247	0.079	0.493	−0.046	0.252	−0.078
Occupational activity	0.261	0.076	0.129	0.052	0.590	0.037

Statistical significance for *p* < 0.05; Spearman’s rank—Spearman’s rank correlation coefficient; prepandemic group—participants tested before the pandemic (December 2019 to March 2020); COVID 1 group—participants tested in the early stages of the pandemic (May to September 2020); COVID 2 group—participants tested in the late stages of the pandemic (June to September 2021).

**Table 7:** Spearman’s Rank Correlations—Physical Activity and Stress.

Level of Perceived Stress						
Group	Prepandemic Group		COVID 1 Group		COVID 2 Group	
Parameters	*p*	Spearman’s Rank	*p*	Spearman’s Rank	*p*	Spearman’s Rank
Total physical activity	0.469	0.049	0.060	0.126	0.943	−0.005
Sports activity and physical exercise	0.048	−0.134	0.059	−0.127	0.317	−0.068
Passive rest	0.322	0.067	0.660	−0.030	0.996	0.000
Movement	0.580	0.038	0.287	0.072	0.837	−0.014
Household activities	0.966	0.003	0.026	0.149	0.371	0.061
Occupational activity	0.823	0.015	0.325	−0.066	0.123	−0.105

Statistical significance for *p* < 0.05; Spearman’s rank—Spearman’s rank correlation coefficient; prepandemic group—participants tested before the pandemic (December 2019 to March 2020); COVID 1 group—participants tested in the early stages of the pandemic (May to September 2020); COVID 2 group—participants tested in the late stages of the pandemic (June to September 2021).

**Table 8:** Regression Analysis.

Variable	Compared Categories	Total Physical Activity [MET-h/Week]	Sports Activity and Physical Exercise [MET-h/week]	Passive Rest [MET-h/Week]	Movement [MET-h/Week]	Household Activities [MET-h/Week]	Occupational Activity [MET-h/Week]
		β (95% CI)	β (95% CI)	β (95% CI)	β (95% CI)	β (95% CI)	β (95% CI)
Group	COVID 1 vs. Prepandemic						
	Model 1 ^#^	−19.1 (−36.8; −1.4) ***	0.7 (−1.3; 2.6)	−3.2 (−7.1; 0.7)	−5.5 (−9.3; −1.7) *	−7.1 (−21.0; 6.8)	−1.4 (−19.4; 16.6)
	Model 2 ^##^	−21.0 (−38.1; −3.9) ***	1.1 (−0.8; 3.0)	−0.9 (−4.5; 2.8)	−5.2 (−9.0; −1.4) *	−14.6 (−26.9; −2.3) ***	4.0 (−14.0; 21.9)
	Model 3 ^###^	−17.7 (−35.2; −0.3) ***	1.1 (−0.9; 3.1)	0.1 (−3.5; 3.7)	−4.3 (−8.2; −0.5) ***	−14.4 (−27.1; −1.8) ***	2.4 (−16.4; 21.2)
	COVID 2 vs. Prepandemic						
	Model 1 ^#^	−32.9 (−50.7; −15.1) ***	0.6 (−1.4; 2.5)	−4.1 (−8.1; −0.2) ***	−2.6 (−6.4; 1.2)	−24.3 (−38.3; −10.3) ***	−5.4 (−22.8; 12.1)
	Model 2 ^##^	−29.4 (−46.6; −12.1) ***	0.7 (−1.2; 2.7)	−3.0 (−6.6; 0.7)	−2.1 (−5.9; 1.7)	−24.6 (−37.1; −12.2) ***	−0.8 (−18.3; 16.6)
	Model 3 ^###^	−26.1 (−43.7; −8.5) *	0.6 (−1.4; 2.6)	−2.0 (−5.7; 1.7)	−1.1 (−4.9; 2.8)	−24.1 (−36.8; −11.4) ***	−0.9 (−19.2; 17.4)
SWLS	per 1 unit						
	Model 1 ^#^	0.5 (−1.2; 2.2)	0.1 (0.0; 0.3)	−0.6 (−1.0; −0.3) ***	0.1 (−0.2; 0.5)	0.4 (−0.9; 1.7)	−0.1 (−1.9; 1.8)
	Model 2 ^##^	1.0 (−0.7; 2.6)	0.1 (0.0; 0.3)	−0.6 (−0.9; −0.2) *	0.2 (−0.2; 0.5)	0.5 (−0.7; 1.7)	0.0 (−1.8; 1.9)
	Model 3 ^###^	1.1 (−0.6; 2.8)	0.1 (0.0; 0.3)	−0.6 (−0.9; −0.2) *	0.3 (−0.1; 0.6)	0.5 −0.7; 1.7)	0.5 (−1.5; 2.4)
Stress	per 1 unit						
	Model 1 ^#^	1.3 (0.1; 2.5) ***	−0.1 (−0.2; 0.1)	0.0 (−0.3; 0.3)	0.2 (0.0; 0.5)	1.1 (0.2; 2.1) ***	0.1 (−1.1; 1.4)
	Model 2 ^##^	1.1 (−0.1; 2.3)	0.0 (−0.2; 0.1)	0.2 (−0.1; 0.4)	0.2 (0.0; 0.5)	0.6 (−0.2; 1.4)	0.2 (−1.0; 1.4)
	Model 3 ^###^	1.1 (0.0; 2.3)	0.0 (−0.2; 0.1)	0.2 (−0.1; 0.4)	0.3 (0.0; 0.5) ***	0.5 (−0.3; 1.4)	0.5 (−0.8; 1.8)
Marital status	Unmarried vs. Married						
	Model 1 ^#^	-	-	-	-	-	-
	Model 2 ^##^	49.3 (29.1; 69.6) ***	0.4 (−1.9; 2.7)	7.8 (3.5; 12.1) ***	7.4 (2.9; 11.9) *	14.9 (0.3; 29.6) ***	23.4 (5.9; 40.9) *
	Model 3 ^###^	42.4 (20.3; 64.6) ***	0.8 (−1.8; 3.3)	4.6 (0.0; 9.2) *	7.7 (2.9; 12.6) *	11.7 (−4.3; 27.7)	15.6 (−4.1; 35.3)
Place of residence	City < 100,000 vs. City > 100,000						
	Model 1 ^#^	-	-	-	-	-	-
	Model 2 ^##^	22.3 (−0.5; 45.1)	−2.1 (−4.7; 0.5)	−3.9 (−8.8; 1.0)	2.7 (−2.3; 7.8)	19.9 (3.5; 36.3) ***	5.4 (−15.9; 26.6)
	Model 3 ^###^	16.7 (−6.7; 40.1)	−2.2 (−4.9; 0.4)	−4.9 (−9.8; −0.1) ***	1.7 (−3.4; 6.9)	17.8 (0.9; 34.7) ***	1.7 (−21.0; 24.3)
	Village vs. City > 100,000						
	Model 1 ^#^	-	-	-	-	-	-
	Model 2 ^##^	6.3 (−10.1; 22.6)	−2.9 (−4.8; −1.1) *	−5.4 (−8.9; −1.9) *	0.3 (−3.3; 3.9)	18.2 (6.4; 30.0) *	−11.6 (−29.3; 6.2)
	Model 3 ^###^	1.3 (−15.7; 18.4)	−2.9 (−4.8; −0.9) *	−5.8 (−9.4; −2.3) *	−1.2 (−5.0; 2.5)	14.3 (2.0; 26.6) ***	−11.4 (−29.8; 7.1)
Number of deliveries	Multiparous vs. Primiparous						
	Model 1 ^#^	-	-	-	-	-	-
	Model 2 ^##^	48.2 (33.9; 62.5) ***	−2.6 (−4.2; −1.0)*	−14.4 (−17.4; −11.3) ***	1.3 (−1.9; 4.4)	69.5 (59.2; 79.8) ***	−2.4 (−17.1; 12.3)
	Model 3 ^###^	53.7 (37.8; 69.6) ***	−2.2 (−4.0; −0.4) ***	−14.7 (−18.0; −11.3) ***	2.7 (−0.8; 6.2)	74.7 (63.2; 86.2) ***	−2.6 (−20.1; 14.9)

*** *p* < 0.001, * *p* < 0.05; ^#^ Model 1 includes: group (COVID 1/COVID 2/prepandemic (ref.)), SWLS (score), stress (score); ^##^ Model 2 includes: group (COVID 1/COVID 2/prepandemic (ref.)), SWLS (score), stress (score), marital status (unmarried/married (ref.)), place of residence (village/city < 100,000/city > 100,000 (ref.)), number of deliveries (multiparous/primiparous (ref.)), education (primary or vocational or secondary/university (ref.)), planning the current pregnancy (yes/no (ref.)), complications in pregnancy(no/yes (ref.)); ^###^ Model 3 includes: group (COVID 1/COVID 2/prepandemic (ref.)), SWLS (score), stress (score), marital status (unmarried/married (ref.)), place of residence (village/city < 100,000/city > 100,000 (ref.)), number of deliveries (multiparous/primiparous (ref.)), education (primary or vocational or secondary/university (ref.)), planning the current pregnancy (yes/no (ref.)), complications in pregnancy(no/yes (ref.)), age (years), delivery at term (yes/no (ref.)), type of delivery (cesarean section/vaginal delivery (ref.)).

## Data Availability

The data underlying this article will be shared on reasonable request to the corresponding author.
